# Hospital mortality prediction in traumatic injuries patients: comparing different SMOTE-based machine learning algorithms

**DOI:** 10.1186/s12874-023-01920-w

**Published:** 2023-04-22

**Authors:** Roghayyeh Hassanzadeh, Maryam Farhadian, Hassan Rafieemehr

**Affiliations:** 1 Department of Biostatistics, School of Public Health, Hamadan University of Medical Sciences, Hamadan, Iran; 2 Research Center for Health Sciences, Department of Biostatistics, School of Public Health, Hamadan University of Medical Sciences, Hamadan, Iran; 3 Department of Medical Laboratory Sciences, School of Paramedicine, Hamadan University of Medical Sciences, Hamadan, Iran

**Keywords:** Imbalanced data, Machine learning algorithms, SMOTE family techniques, Traumatic injuries

## Abstract

**Background:**

Trauma is one of the most critical public health issues worldwide, leading to death and disability and influencing all age groups. Therefore, there is great interest in models for predicting mortality in trauma patients admitted to the ICU. The main objective of the present study is to develop and evaluate SMOTE-based machine-learning tools for predicting hospital mortality in trauma patients with imbalanced data.

**Methods:**

This retrospective cohort study was conducted on 126 trauma patients admitted to an intensive care unit at Besat hospital in Hamadan Province, western Iran, from March 2020 to March 2021. Data were extracted from the medical information records of patients. According to the imbalanced property of the data, SMOTE techniques, namely SMOTE, Borderline-SMOTE1, Borderline-SMOTE2, SMOTE-NC, and SVM-SMOTE, were used for primary preprocessing. Then, the Decision Tree (DT), Random Forest (RF), Naive Bayes (NB), Artificial Neural Network (ANN), Support Vector Machine (SVM), and Extreme Gradient Boosting (XGBoost) methods were used to predict patients' hospital mortality with traumatic injuries. The performance of the methods used was evaluated by sensitivity, specificity, Positive Predictive Value (PPV), Negative Predictive Value (NPV), accuracy, Area Under the Curve (AUC), Geometric Mean (G-means), F1 score, and *P*-value of McNemar's test.

**Results:**

Of the 126 patients admitted to an ICU, 117 (92.9%) survived and 9 (7.1%) died. The mean follow-up time from the date of trauma to the date of outcome was 3.98 ± 4.65 days. The performance of ML algorithms is not good with imbalanced data, whereas the performance of SMOTE-based ML algorithms is significantly improved. The mean area under the ROC curve (AUC) of all SMOTE-based models was more than 91%. F1-score and G-means before balancing the dataset were below 70% for all ML models except ANN. In contrast, F1-score and G-means for the balanced datasets reached more than 90% for all SMOTE-based models. Among all SMOTE-based ML methods, RF and ANN based on SMOTE and XGBoost based on SMOTE-NC achieved the highest value for all evaluation criteria.

**Conclusions:**

This study has shown that SMOTE-based ML algorithms better predict outcomes in traumatic injuries than ML algorithms. They have the potential to assist ICU physicians in making clinical decisions.

**Supplementary Information:**

The online version contains supplementary material available at 10.1186/s12874-023-01920-w.

## Introduction

Trauma is one of the world's most critical public health issues, leading to death and disability and influencing all age groups [1]. Traumatic injuries are the leading cause of mortality in the first four decades of life [2]. Trauma causes 4.4 million deaths annually and accounts for almost 8% of all deaths worldwide [1, 3]. In this regard, it is important to find solutions to reduce the impact of traumatic injuries and the number of deaths resulting from trauma. For example, improving the ability to predict the outcome of a trauma patient with a high degree of accuracy and identifying important factors that influence the patient's outcome can assist medical trauma teams in their rapid efforts to treat trauma patients.

Many previous studies have used traditional methods such as the logistic and Poisson regression models to identify factors that influence traumatic injuries [4–6]. Numerous studies have also used the Trauma and Injury Severity Score (TRISS) as one of the most common models, which is based on logistic regression (LR) and uses a small cohort from a single center to predict the probability of survival of patients with traumatic injuries [7]. However, the TRISS and its various modifications are evidence-based tools, and the results of some studies indicate that they may mislead physicians by misclassifying the patient's condition [8]. Nevertheless, both categories of models performed poorly when collinearity, heteroskedasticity, higher order interactions, and nonlinear relationships among variables were present [9–11]. Hence, more valuable and accurate prognostic tools that are not limited to these assumptions are needed to achieve better patient outcomes and make the best use of resources.

In recent decades, methods based on machine learning algorithms have been developed whose main advantage is that they overcome the problems of classical methods [12, 13]. Recently, various ML methods have been used to predict outcomes in medical research, especially in trauma [14–19]. In addition, several studies have compared the performance of ML methods with evidence-based and regression models such as TRISS for predicting mortality in trauma patients [11, 17].

However, ML algorithms may be inappropriate when they encounter imbalanced data. An imbalanced data set is common in medical data. It occurs when there are many more instances of one class (majority class) than the other class (minority class). In such cases, the predictive ability of the classifiers is impaired because they are biased towards the majority classes and misclassify the minority class instances. Consequently, the classifiers provide high predictive accuracy for the majority class. Therefore, if the data are imbalanced, the criterion of accuracy is not suitable to evaluate the performance of the classifiers. Although, the minority class is often the main class that researchers want to predict with higher accuracy [20–22]. Nevertheless, the problem of imbalanced data is critical, but investigations have shown that less attention has been paid to this problem in recent studies. For trauma, the data are generally unbalanced. Nevertheless, the results of a recent systematic review in this area show that most studies support the benefits of ML models [23]. However, the sensitivity–specificity gap values showed a wide range (0.035 to 0.927), highlighting the risk of imbalanced data [10, 23].

There are several methods to deal with the imbalanced class, such as resampling data by oversampling or under-sampling, increasing the cost of the minority class classification error, or learning only one class [21, 24, 25]. The synthetic minority oversampling (SMOTE) method proposed by Chawla et al. is the first model in the SMOTE family to be widely used in imbalance problems [21]. Over time, many SMOTE algorithms have been proposed, such as borderline SMOTE, ADASYN, SMOTE-NC, and SVM-SMOTE [26].

To the best of our knowledge, most of the studies conducted have evaluated the performance of SMOTE techniques using simulated data and publicly available data [27–29]. Moreover, few studies have used these techniques in trauma, and there is no study that has addressed in depth the prediction of traumatic injury in Iran. In this work, five SMOTE methods, such as SMOTE, Borderline-SMOTE1, Borderline-SMOTE2, SMOTE-NC, and SVM-SMOTE, were used to balance imbalanced datasets. We selected these methods among the numerous SMOTE variants because they belong to the category of data-level techniques that can be flexibly combined with other methods and are easier to use compared to algorithm-level approaches. Moreover, these methods are more adaptable since their application does not depend on the chosen classifier. They are also the most commonly used resampling methods in the literature [21, 26–28].

Therefore, the main objective of this study is to comprehensively compare the performance of six ML algorithms, namely DT, RF, NB, ANN, XGBoost, and SVM, based on five techniques of the SOMT family for predicting hospital mortality in patients with traumatic injuries. In addition, identify important variables in predicting hospital mortality in patients with traumatic injuries was referred to the Besat hospital of Hamadan city from—March 2020 to—March 2021.

## Materials and methods

### Data collection and preparation

The present study was a retrospective cohort study conducted on 126 trauma patients. These patients were admitted to an intensive care unit at the Besat hospital of Hamadan province, in the west of Iran, from—March 2020 to—March 2021. The data were extracted from the patients’ medical records. Our focus was on the information about trauma patients' status (alive/dead) as a response and related risk factors to trauma. Patients were followed up from the time they entered the ICU until death or discharge, and the mean follow-up time from the date of trauma to the date of outcome was 3.98 days. We chose six risk factors associated with trauma outcome including, age, sex (male, female), type of trauma (blunt, penetrating), location of injuries (head and neck, thorax, abdomen and pelvic, spinal, extremities, multi-injuries), Glasgow coma scale (severe, moderate, minor) and white blood cells (k /mm^3^) to evaluate the performance of ML methods.

#### Decision tree

Decision Tree is one of the easiest and popular algorithms for classification and regression problems. The main goal of the DT is to construct a model that can predict the value of a target variable by learning simple decision rules deduced from the data features. Nodes and branches are the two main components of a DT model. The three essential steps in making a DT model are division, stopping, and pruning. The tree's making starts with all training data in the first node. Then, the first partition splits the data into two or more daughter nodes based on a predictor variable [30].

DT contains three types of nodes. (a) A root node or decision node indicates a decision that will result in the subdivision of all features into two or more mutually exclusive subsets. This node has no input branch, and the number of its output branches can be zero or more. (b) Internal nodes indicate one of the possible selects available in the tree structure; the Input branch of the node is linked to its parent node, and the output branch of the node is linked to its child nodes or leaf nodes. (c) Leaf nodes or terminal nodes indicate the final conclusion of a combination of decisions or events. These have one input branch and no output branch [31].

The benefit of DT contains simplicity in interpretation, the facility to handle categorical and quantitative values, the ability to fill missing values in features with the most probable value, and robustness to outliers. The main drawback of the decision tree is that it can be exposed to overfitting and under-fitting, especially when using a small data set [32].

#### Random forest

The RF method was first proposed by Leo Breiman [33]. This algorithm is an ensemble learning method used widely in classification and regression problems. It produces a large number of decision trees from subsamples of the dataset. Each decision tree will generate an output. Then the final output is obtained based on majority votes for classification and the average for regression. At first, in this algorithm, bootstrap samples were drawn through the resampling of the original data. Approximately 37% of the data is excluded from each bootstrap sample, named out-of-bag or OOB data. Afterward, for each of the bootstrap samples, RF will create an unpruned tree as follow: At each tree node, some variables were randomly picked from all variables, and then picked the best split from among those variables. All the decision data created from the bootstrap samples are compounded and analyzed to gain the final RF model [13, 33].

The performance of the random forest can be estimated by its internal validation using the OOB data. For classification issues, the RF's classification error rate, which is named out-of-bag (OOB) error will be calculated from OOB data. Each bootstrap iteration will be predicted using the tree grown with the bootstrap sample for the OOB data. Then will be cumulated the OOB predictions and computed the error rate or OOB error [34]. A benefit of the OOB error is that original data is used for its estimation and the other benefit of using it is high computational speed [35]. Many studies represent that the RF algorithm compared with other ML algorithms has higher stability, robustness and high classification performance. Also, it can preserve high classification performance when missing data exist [18]. Another property of the RF method is the generation of prediction rules. This method can identify essential variables [13].

#### Naïve bayes

The NB classifier is a simple algorithm that applies the famous Bayes’ theorem with strong independence assumptions. Indeed, the NB classifier supposes that all predictor variables are conditionally independent of one another. NB method looks for a clear, simple, and very quick classifier. NB classification model categorized samples by computing the probability that an object belongs to a specific category. Due to the Bayesian formula, the posterior probability is computed according to the prior probability of an object, and the class with the maximum posterior probability is chosen as the object's class. Easy implementation, good performance, working with little training data and making probabilistic predictions are advantages NB method. Also, it is not sensitive to unrelated features. In addition, NB executes well, even when the independence assumption is violated. However, it is computationally intensive, especially for models involving many variables [15, 32].

#### Artificial neural network

An artificial neural network inspired by the operation of neurons in the human brain is a machine learning method widely used that performs mightily in classification and pattern identification. The learning process in this method performs via gathering information by detecting patterns and relationships in data and learning through experience. A multilayer feed-forward neural network consists of an input layer, one or more hidden layers, and an output layer. The hidden layer is intermediate between the input and output layers, and the number is commonly specified with the cross-validation method. Each layer is made up of units called neurons (nodes). The neurons in the two adjacent layers are fully connected in which each connection has a weight associated with it, while the neurons inside the same layer are not connected. In the feed-forward neural network, information proceeds unidirectionally. Information traverses from the input layer neurons and transits from the hidden layer's neurons to the output neurons. Furthermore, in a neural network, complex non-linear mappings between input and output are taught by activation functions [13, 32]. In this study, we used the sigmoid activation function because it is a non-linear activation function usually used before the output layer in binary classification.

### Support vector machine

The SVM is based on statistical learning theory and was first suggested by Vapnik [36]. The main aim of SVM is to find a particular linear model that maximizes hyper-plane margin. Maximizing the hyper-plane margin will maximize the distance between classes. The nearest training points to the maximum cloud margin are the support vectors. Hence, classification is performed by mapping a vector of variables into a high-dimensional plane by maximizing the margin between two data classes. The SVM algorithm can classify both linear and nonlinear observations. When data are not linearly separable, SVM using a kernel function transforms nonlinear input to a linear state in high-dimensional feature space and carries out the linear separation in this new space. In order to do this, several kernel functions have been proposed and adopted for SVM, such as linear, radial, polynomials, and sigmoid [13]. Selecting the kernel function in the SVM makes it a flexible method [9]. In the present study, we employed the radial basis kernel function for its better performance.

### Extreme gradient boosting

XGBoost algorithm has gradient boosting at its core but is an enhanced version of the gradient-boosted decision tree algorithm. This algorithm is a scalable tree-boosting system to overcome long learning times, and Chen and Guestrin developed the overfitting of traditional boosting algorithms in 2016 [37]. XGBoost classifier synthesizes a weak base classifier with a robust classifier. A base classifier’s residual error is utilized in the next classifier to optimize the objective function at each stepwise of the training process [38]. Moreover, this algorithm can restrict overfitting, decrease classification errors, handle the missing values and minimize learning times while developing the final model [39].

### SHAP value

Machine learning models have great potential in prediction and classification. However, understanding the complexity of the predictive models' results is slightly complicated, which is a barrier to the admission of ML models. Hence to overcome this problem, Lundberg and Lee proposed a novel Shapley additive explanations (SHAP) approach for interpreting predictions for different techniques, including XGBoost. It helps us to describe the prediction of a specific input by calculating the impact of each feature on the prediction. SHAP values obtain interpretability through summary plots and the global importance of the variable [19].

### Synthetic Minority Over-Sampling Technique (SMOTE)

The imbalanced dataset classification problem occurs when the number of instances of one class is greater than that of the other class. In classification problems with two classes, the class with more specimens is named the majority class, and the class with a smaller number of specimens is called the minority class [20]. The level of class imbalance of a dataset is measured by the imbalance ratio (IR). The IR is defined as the ratio of the number of samples in the majority class to the number of samples in the minority class. The higher the IR, the greater the imbalance [40]. In such cases, reporting the prediction accuracy as an evaluation criterion is inappropriate, as this usually leads to a bias in favor of the majority class [21].

Two main approaches have been proposed to solve the class imbalance problem: a data-level approach and an algorithm-based approach. The data-level approach aims to change or modify the class distribution in the dataset before training a classifier, which is usually done in the preprocessing phase. The algorithm-level approach focuses on improving the current classifier by adapting the algorithms to learn minority classes [41].

The data-level approach is usually preferred and proposed to deal with unbalanced classes in classification problems. This could be due to the fact that the class composition of the data can be adjusted to a "relatively balanced" ratio by adding or removing any number of class instances in the data set, depending on the situation [42].

Other reasons that can be given are: 1) The samples generated by these methods represent the right trade-off between introducing variance and approximating the original distribution. 2) These techniques are easier to apply compared to algorithm-level methods because the datasets are cleaned before they are used to train different classifiers. 3) Data-level techniques can be flexibly combined with other methods [26–28].

Re-sampling or data synthesis is the most popular method of processing unbalanced datasets used for data-level approach. The re-sampling approach can be divided into three categories, (i) over-sampling (ii) under-sampling (iii) hybrid sampling [43]. In over-sampling, the weight of the minority class is increased by repeating or generating new samples of the minority class. Under-sampling randomly deletes instances from the majority class to balance with the minority class. Hybrid sampling combines these two methods to take advantage of the benefits and drawbacks of both approaches [43]. The over-sampling approach is generally applied more frequently than other approaches. This approach is called SMOTE family and a collection of numerous over-sampling techniques (85 variants) evolved from SMOTE [26]. One of the first Over-sampling methods, SMOTE, is a powerful tool for dealing with imbalanced data sets suggested by Chawla et al. [21]. SMOTE is an oversampling technique that generates synthetic data for a minority class based on its k-nearest neighbor until the ratio of minority and majority classes becomes more balanced. The new synthetic data are very similar to the actual data because they are produced based on initial features [21].

The main advantage of SMOTE is that it prevents overfitting by synthesizing new samples from the minority class instead of repeating them [44].

There are also some disadvantages of SMOTE, however: oversampling of noisy samples, Oversampling of borderline samples [28]. To overcome these problems, many strategies have been employed in the literature including [28]:

- Extensions of SMOTE by combining it with other techniques such as noise filtering, e.g., SMOTE-IPF and SMOTE-LOF
- Modifications of SMOTE, e.g., borderline SMOTE (B1-SMOTE and B2-SMOTE) and SVM-SMOTE.

Borderline-SMOTE is an extension of SMOTE with a more powerful performance ability proposed by Han et al. in 2005. In this method, only the borderline examples of the minority class are over-sampled. A Borderline is a region where the samples of minority classes are near the majority. At first, the number of majority neighbors of each minority instance is used to split minority instances into three groups: safe, noise, and danger, then generate new instances. Suppose the neighbors of the points in the danger region are considered from the minority class. In that case, this method is called Borderline-SMOTE1, and when the point's neighbors in the danger region are considered from the minority and majority classes, called Borderline-SMOTE2 [45]. Support vector machine SMOTE (SVM-SMOTE) is another extension of SMOTE that generates new synthetic samples near the decision boundary. This approach used SVM to detect decision boundaries [46]. SMOTE-Nominal Continuous (SMOTE-NC) is an over-sampling method that uses k-nearest neighbors, applying the modified-Euclidean distances to generate new synthetic samples [21]. This study introduced SMOTE techniques that have been used in the preparation initial data stage, then training ML algorithms have performed.

### Performance criteria

The predictive performance of ML algorithms was evaluated using several criteria, including sensitivity, specificity, Positive Predictive Value (PPV), Negative Predictive Value (NPV), accuracy, Area Under the Curve (AUC), Geometric Mean (G-means), F1 score, and *P*-value of the McNemar test. We evaluated the predictive performance of ML methods using a cross-validation approach in which both groups of datasets, the original imbalanced dataset, and the SMOTE-balanced datasets, were randomly split into training (70%) and test (30%) sets. This process was iterated 100 times. Then, mean values for each evaluation criterion were calculated over 100 repetitions. Moreover, to prevent over-fitting, ML algorithms performed fivefold cross-validation to select the optimum hyperparameters. Different values for each of hyperparameters were examined and optimum value was determined. The optimal values of hyperparameters selected for each of the ML models are shown in Table 1.

**Table 1:** The tuning parameter values of SMOTE-based machine learning methods

**Methods**	**Hyperparameters**	**Definition**	**Value**
**ANN**	*Size*	The number of nodes in the hidden layer	5
	*Weight decay*	The regularization parameter to avoid overfitting	0.1
**SVM**	*Gamma*	The width of the radial basis function kernel	0.12
	*Cost*	The parameter that controls the complexity of the model	1
**RF**	*mtry*	Number of variables randomly selected as candidates for each tree	2
	*ntree*	The number of trees	500
**DT**	*minsplit*	The minimum number of observations in a node	10
	*minbucket*	The minimum number of observations in any terminal node	3
**XGBoost**	*nrounds*	The maximum number of iterations	100
	*eta*	Learning rate	0.3
	*gamma*	Regularization parameter to prevent overfitting	5
	*max depth*	The depth of the tree	3

### Software packages

In the present study, all SMOTE-balancing methods were executed through programming in Python software version 3.10.6 with the package "imbalanced-learn." Also, all analyses of ML methods were implemented using R software version 4.1.1, with the following packages: “*e1071*” for SVM; “*nnet*” for NN; “*naivebayes*” for NB; “*randomForest*” for RF and variable importance (VIMP) in the RF; “*rpart*” for DT; “*xgboost*” for XGBoost; and “*SHAPforxgboost*” for SHAP value.

## Results

In this study, of the 126 patients admitted to an intensive care unit, 117 (92.9%) were alive and 9 (7.1%) were dead. The mean follow-up time from the date of trauma to the date of outcome was 3.98 ± 4.65 days, with a mean follow-up time of 1.56 ± 0.73 days for patients who died and 4.17 ± 4.77 days for patients who survived. The overall mean (± SD) age of patients with traumatic injuries was 37.71 ± 12.78 years, with a minimum and maximum of 18 and 60 years, respectively. The characteristics of patients according to their traumatic injuries are listed in Table 2. Most of them were men, 85 (67.5%). The mean WBC value of the alive patients (9066.67 ± 2938.57) was significantly lower than that of the dead patients (15,500 ± 4492.22) (*p* < 0.001). Univariate analysis based on the chi-square test showed that the type of trauma in patients and the GCS were significantly related to the outcome of traumatic injuries. Mortality was significantly higher among penetrating trauma (18.5%) than in blunt trauma (4%) (*p* = 0.022). In patients with severe GCS (50%), mortality was significantly higher than in patients with moderate and minor GCS (8.5%) (*p* < 0.001).

**Table 2:** Demographic and clinical characteristics of patients according to traumatic injuries

Variable	Traumatic Injuries Outcome				Total		
	Alive		Dead				
	N	%	N	%	N	%	*P*-value^a^
All	117	92.9	9	7.1	126	100	
Sex							
Male	79	92.9	6	7.1	85	67.4	0.609
Female	38	92.7	3	7.3	41	32.6	
Type of trauma							
Blunt	95	96	4	4	99	78.6	0.022
Penetrating	22	81.5	5	18.5	27	21.4	
Location of injuries							
Head and Neck	33	97.1	1	2.9	34	27	0.175
Thorax	12	100	0	0	12	9.5	
Abdomen and Pelvic	4	100	0	0	4	3.2	
Spinal	3	100	0	0	3	2.4	
Extremities	16	100	0	0	16	12.7	
Multi injuries	49	86	8	14	57	45.2	
GCS							
Minor	79	100	0	0	79	62.7	0.001
Moderate	32	91.4	3	8.6	35	27.8	
Severe	6	50	6	50	12	9.5	
Age(year): Mean ± SD	37.52 ± 12.76		40.22 ± 13.48		*p*-value ^b^	0.543	
LOS (day): Mean ± SD	4.17 ± 4.77		1.56 ± 0.73		*p*-value ^c^	0.105	
WBC: Mean ± SD	9066.67 ± 2938.57		15,500 ± 4492.22			< 0.001	

*LOS* Length of stay in ICU, *WBC* White blood cells, *GCS* Glasgow coma scale, *SD* Standard deviation

^a^Chi-square test

^b^T-test

^c^Mann-Whitney Test

According to the findings, the ratio of dead to alive population was 1:13 (IR = 13), expressing an extreme imbalance between the two classes. Therefore, various SMOTE family techniques were applied to face the imbalance of the data in the original datasets.

Initially, all classifiers are performed on the imbalanced data to represent the impact of the imbalanced data problem on the performance of the classifiers. Afterward, all classifiers are conducted on balanced data generated by SMOTE family techniques.

Table 3 demonstrates the performance of the six ML algorithms for the prediction of mortality in patients with traumatic injuries on the imbalanced datasets (original) and on the balanced dataset in terms of sensitivity, specificity, PPV, NPV, accuracy, AUC, G-means, F1-score, and *P*-value of McNemar's test. Further details on the 95% confidence intervals for each criterion of the models used are provided in Additional file 1.

**Table 3:** Comparison of the prediction performances of SMOTE-based machine learning methods on the test dataset

Methods	Dataset	Sensitivity	Specificity	PPV	NPV	Accuracy	AUC	G-means	F1-Score	McNemar
		**Mean (SE)**	**Mean (SE)**	**Mean (SE)**	**Mean (SE)**	**Mean (SE)**	**Mean (SE)**	**Mean (SE)**	**Mean (SE)**	**Mean (SE)**
**SVM**	Original	0.55(0.0469)	**1(0)**	**1(0)**	0.96(0.0034)	0.97(0.0037)	0.72(0.0095)	0.66(0.0150)	0.60(0.0192)	0.75(0.0165)
	SMOTE	0.98(0.0009)	0.97(0.0018)	0.97(0.0018)	**0.99(0.0001)**	0.99(0.0011)	0.98(0.0009)	0.98(0.0009)	0.98(0.0009)	0.26(0.0251)
	B-SMOTE1	**0.99(0.0011)**	0.96(0.0019)	0.96(0.0019)	0.99(0.0014)	0.98(0.0011)	0.98(0.0011)	0.98(0.0011)	0.98(0.0011)	0.74(0.0098)
	B-SMOTE2	0.95(0.0035)	0.96(0.0029)	0.96(0.0030)	0.95(0.0037)	0.95(0.0020)	0.95(0.0019)	0.95(0.0020)	0.95(0.0019)	0.53(0.0322)
	SMOTE-NC	0.98(0.0004)	0.99(0.0001)	0.99(0.0001)	0.98(0.0005)	**0.99(0.0004)**	**0.99(0.0002)**	**0.99(0.0002)**	**0.99(0.0002)**	0.33(0.0096)
	SVM-SMOTE	0.99(0.0012)	0.96(0.0024)	0.94(0.0040)	0.99(0.0010)	0.98(0.0015)	0.98(0.0012)	0.98(0.0012)	0.97(0.0021)	0.73(0.0122)
**ANN**	Original	0.78(0.7474)	**1(0)**	0.76(0.0203)	0.97(0.0051)	0.98(0.0217)	0.89(0.1182)	0.81(0.0223)	0.69(0.0246)	0.26(0.816)
	SMOTE	**1(0)**	**1(0)**	**1(0)**	**1(0)**	**1(0)**	**1(0)**	**1(0)**	**1(0)**	NA
	B-SMOTE1	**1(0)**	**1(0)**	**1(0)**	**1(0)**	**1(0)**	**1(0)**	**1(0)**	**1(0)**	NA
	B-SMOTE2	0.98(0.0014)	0.98(0.0018)	0.98(0.0018)	0.98(0.0013)	0.99(0.0016)	0.98(0.0012)	0.98(0.0012)	0.98(0.0013)	0.21(0.0249)
	SMOTE-NC	0.97(0.0016)	0.98(0.0012)	0.98(0.0013)	0.98(0.0015)	0.99(0.0015)	0.98(0.0012)	0.98(0.0012)	0.98(0.0013)	0.20(0.0232)
	SVM-SMOTE	0.98(0.0003)	0.99(0.0002)	0.98(0.0003)	0.99(0.0001)	0.99(0.0002)	0.98(0.0001)	0.98(0.0001)	0.98(0.0003)	0.33(0.0031)
**NB**	Original	0.51(0.0491)	0.95(0.0048)	0.42(0.0324)	0.96(0.0037)	0.91(0.0064)	0.70(0.0241)	0.66(0.0170)	0.44(0.0190)	0.53(0.0337)
	SMOTE	**0.99(0.0004)**	0.91(0.0052)	0.92(0.0046)	**0.99(0.0005)**	0.95(0.0026)	0.95(0.0026)	0.95(0.0028)	0.96(0.0025)	0.87(0.0148)
	B-SMOTE1	0.99(0.0012)	0.92(0.0046)	0.93(0.0042)	0.99(0.0016)	0.96(0.0024)	0.96(0.0023)	0.96(0.0024)	0.96(0.0023)	0.85(0.0111)
	B-SMOTE2	0.96(0.0036)	0.86(0.0059)	0.87(0.0055)	0.96(0.0040)	0.91(0.0035)	0.91(0.0034)	0.91(0.0035)	0.91(0.0034)	0.75(0.0293)
	SMOTE-NC	0.98(0.0019)	**0.95(0.0032)**	**0.95(0.0032)**	0.98(0.0020)	**0.96(0.0018)**	**0.96(0.0018)**	**0.96(0.0019)**	**0.96(0.0019)**	0.61(0.0282)
	SVM-SMOTE	0.99(0.0012)	0.91(0.0050)	0.88(0.0063)	0.99(0.0079**)**	0.94(0.0031)	0.95(0.0025)	0.95(0.0028)	0.93(0.0036)	0.86(0.0129)
**RF**	Original	0.53(0.0348)	0.99(0.0005)	0.98(0.0222)	0.95(0.0041)	0.96(0.0039)	0.71(0.0122)	0.65(0.0176)	0.60(0.0224)	0.76(0.0230)
	SMOTE	**0.99(0.0003)**	**0.99(0.0001)**	**0.99(0.0001)**	**0.99(0.0003)**	**0.99(0.0003)**	**0.99(0.0001)**	**0.99(0.0001)**	**0.99(0.0002)**	0.07(0.0062)
	B-SMOTE1	0.98(0.0007)	0.99(0.0002)	0.99(0.0002)	0.98(0.0010)	0.99(0.0005)	0.98(0.0003)	0.99(0.0004)	0.99(0.0003)	0.08(0.0109)
	B-SMOTE2	0.97(0.0025)	0.98(0.0021)	0.97(0.0023)	0.97(0.0025)	0.98(0.0019)	0.97(0.0015)	0.97(0.0015)	0.97(0.0016)	0.38(0.0293)
	SMOTE-NC	0.97(0.0017)	0.98(0.0014)	0.98(0.0015)	0.97(0.0017)	0.99(0.0015)	0.98(0.0012)	0.98(0.0012)	0.98(0.0013)	0.25(0.0254)
	SVM-SMOTE	0.97(0.0018)	0.99(0.0002)	0.98(0.0002)	0.98(0.0012)	0.99(0.0010)	0.98(0.0009)	0.98(0.0009)	0.97(0.0010)	0.25(0.017)
**DT**	Original	0.26(0.5799)	0.94(0.0046)	0.14(0.0295)	0.96(0.0045)	0.90(0.0061)	0.60(0.0282)	0.45(0.0135)	0.25(0.0086)	0.35(0.0583)
	SMOTE	0.94(0.0059)	0.92(0.0058)	0.92(0.0055)	0.94(0.0052)	0.93(0.0035)	0.93(0.0035)	0.93(0.0036)	0.93(0.0037)	0.65(0.0322)
	B-SMOTE1	0.93(0.0048)	0.93(0.0057)	0.93(0.0054)	0.93(0.0047)	0.93(0.0033)	0.93(0.0032)	0.93(0.0033)	0.93(0.0032)	0.64(0.0303)
	B-SMOTE2	0.92(0.0067)	0.91(0.0076)	0.91(0.0072	0.93(0.0060)	0.91(0030)	0.92(0.0028)	0.91(0.0030)	0.91(0.0031)	0.82(0.0242)
	SMOTE-NC	0.94(0.0043)	**0.97(0.0045)**	**0.97(0.0045)**	0.95(0.0038)	**0.96(0.0024)**	**0.96(0.0023)**	**0.96(0.0024)**	**0.96(0.0025)**	0.80(0.0150)
	SVM-SMOTE	**0.95(0.0042)**	0.94(0.0062)	0.91(0.0080)	**0.97(0.0029)**	0.94(0.0041)	0.94(0.0037)	0.94(0.0038)	0.93(0.0048)	0.58(0.0311)
**XGBoost**	Original	0.99(0.0026)	0.45(0.0122)	0.13(0.0070)	0.99(0.0007)	0.49(0.0116)	0.72(0.061)	0.66(0.0109)	0.22(0.0107)	0.99(0.0006)
	SMOTE	0.99(0.0021)	0.96(0.0043)	0.96(0043)	0.98(0.0019)	0.97(0.0021)	0.97(0.0020)	0.97(0.0020)	0.97(0.0021)	0.64(0.0352)
	B-SMOTE1	0.98(0.0026)	0.96(0.0041)	0.96(0.0039)	0.98(0.0029)	0.97(0.0021)	0.97(0.0020)	0.97(0.0021)	0.97(0.0020)	0.64(0.0362)
	B-SMOTE2	0.96(0.0033)	0.94(0.0040)	0.94(0.0042)	0.96(0.0033)	0.95(0.0022)	0.95(0.0021)	0.95(0.0022)	0.95(0.0023)	0.60(0.0314)
	SMOTE-NC	**1(0)**	**1(0)**	**1(0)**	**1(0)**	**1(0)**	**1(0)**	**1(0)**	**1(0)**	NA
	SVM-SMOTE	0.97(0.0057)	0.96(0.0042)	0.94(0.0062)	0.98(0.0038)	0.96(0.0030)	0.96(0.0029)	0.96(0.0031)	0.95(0.0038)	0.67(0.0319)

*SVM* Support vector machine, *ANN* Artificial neural network, *NB* Naïve bayes, *RF* Random forest, *DT* Decision tree, *XGBoost* extreme gradient boosting, *SMOTE* Synthetic minority over-sampling technique, *B-SMOTE1* Borderline-SMOTE1, *B-SMOTE-2* Borderline-SMOTE2, *PPV* Positive predictive value, *NPV* Negative predictive value, *AUC* Area under the curve

One of the most important results from Table 3 is a considerable discrepancy between specificity and sensitivity in all ML methods used before balancing the dataset. In addition, it can be seen in Table 3 that in the rows of the original dataset, all methods used had high accuracy (≥ 90%). In comparison, the sensitivity values for all algorithms except ANN and XGBoost were less than 55%, which means that the classifiers are biased towards the majority class.

The results in Table 3 show that all methods used except XGBoost have high accuracy (≥ 90%) and specificity (≥ 92%) before and after SMOTE techniques. Compared with imbalanced data, the accuracy of the classifiers increases by a maximum of 8% with balanced data. The sensitivity and AUC of all the algorithms used before SMOTE techniques were significantly lower than after SMOTE techniques. The specificity of all models except XGBoost slightly decreased after the application of SMOTE techniques. In five ML Models, namely, SVM, NB, DT, XGBoost, and RF, the sensitivity and ACU were significantly increased by the use of SMOTE techniques, but the ANN model showed a slight increase in these criteria. For example, with imbalanced data, the DT classifier achieved a sensitivity of 26%, while the result with the SVM-SOMTE technique increased to 95%.

Before applying the SMOTE algorithm, the G-means score for DT was 45%, and for the other models, it was between 60 and 81%. After applying the SMOTE algorithm, the G-means score for all models was over 91%.

The F1 score ranged from 60 to 81% when unbalanced data were used, while it increased to exceed 90% for all models after the SMOTE technique was applied.

Among the SMOTE-based data-balancing techniques, the SMOTE-NC technique attained the highest accuracy value for XGBoost (100%) and SVM (99%), NB, and DT (96%), while Borderline-SMOTE1 provided the highest value of 100% for the ANN Model. SMOTE for ANN and RF also obtained an accuracy of 100% and 99%, respectively. Sensitivity was highest for SMOTE to ANN, RF, and NB, with the highest value of 100%, 99%, and 99%, respectively, whereas Borderline-SMOTE1 had the highest value of 100% to ANN and 99% for SVM. XGBoost with SMOTE-NC also yielded a sensitivity of 100%, and DT with SMOTE-SVM yielded a sensitivity of 95%. Three ML models, namely XGBoost, SVM, and DT with SMOTE-NC, achieved specificity and PPV of 100%, 99%, and 97%, respectively. The ANN model for SMOTE and Borderline-SMOTE1 achieved a specificity and PPV of 100%. RF with SMOTE also had both specificity and PPV 99%.

Based on the NPV comparison of ML algorithms, the performance of the ANN, SVM, and RF classifiers using the SMOTE method was 100%, 99%, and 99%, respectively. In addition, SMOTE-NC provided the highest value of 100% for XGBoost, Borderline-SMOTE1 provided the highest value of 100% for ANN, and the SVM-SMOTE method achieved the highest value of 97% for the DT model.

According to AUC, the performance of the XGBoost, SVM, NB, and DT classifiers with the SMOTE-NC method was 100%, 99%, 96%, and 96%, respectively, while Borderline-SMOTE1 gave the highest value of 100% for the ANN Model. ANN and RF classifiers with SMOTE also obtained AUC of 100% and 99%, respectively.

Finally, the *P*-value of McNemar’s test for all classifiers was greater than 0.05. Consequently, there was no significant difference between the frequencies of false positives and false negatives between two classes.

**Fig. 1:**
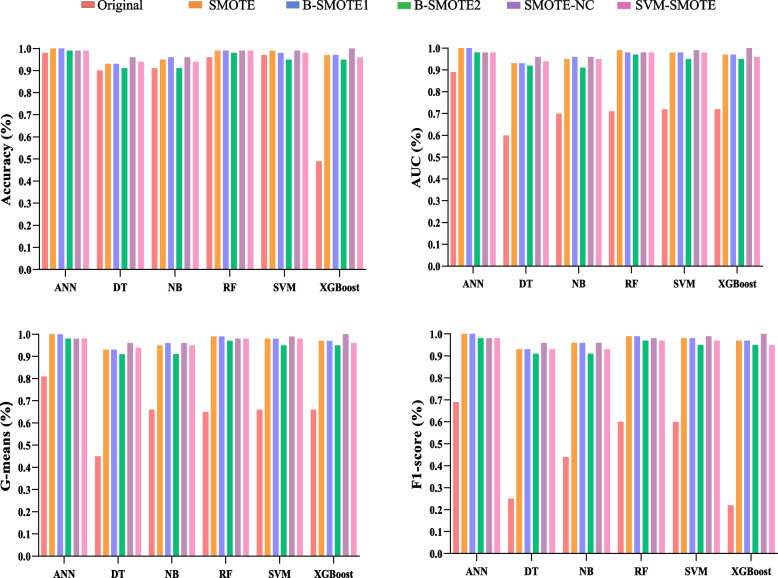
Comparison of the performance of classifiers with SMOTE techniques and without SMOTE in terms of accuracy, AUC, G-means and F1 score

In summary, the SMOTE-NC balancing technique outperformed all other four data balancing techniques based on several evaluation criteria for four classifiers: SVM, NB, DT, and XGBoost. Moreover, the XGBoost model outperformed three other ML models among these ML classifiers. The performance comparison of the classifiers with SMOTE techniques and without SMOTE in terms of accuracy, AUC, G-means, and F1 score is shown in Fig. 1. The plots comparing the performance of the classifiers according to other criteria can be found in Additional file 2.

**Fig. 2:**
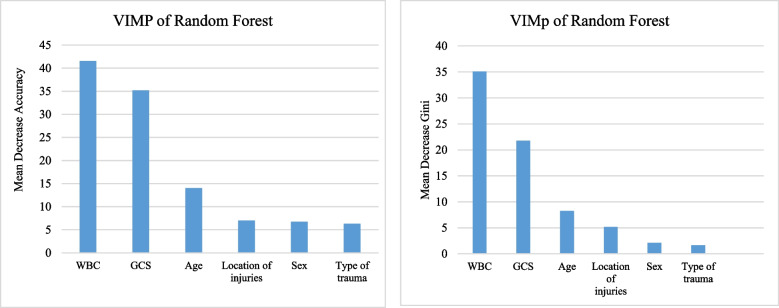
Variable importance from the RF method, in terms of mean decrease accuracy and mean decrease Gini for predicting mortality traumatic injuries patients. WBC: white blood cells, GCS: Glasgow coma scale

According to the SMOTE dataset, the RF model outperformed the other ML methods based on all evaluation criteria. Therefore, Fig. 2 indicates the relative importance of each variable obtained by the RF method in terms of mean decrease accuracy and mean decrease Gini. These indices identified WBC, GCS, and Age as the three most important variables for predicting trauma injury mortality. Afterward, the location of injuries and sex were important variables.

**Fig. 3:**
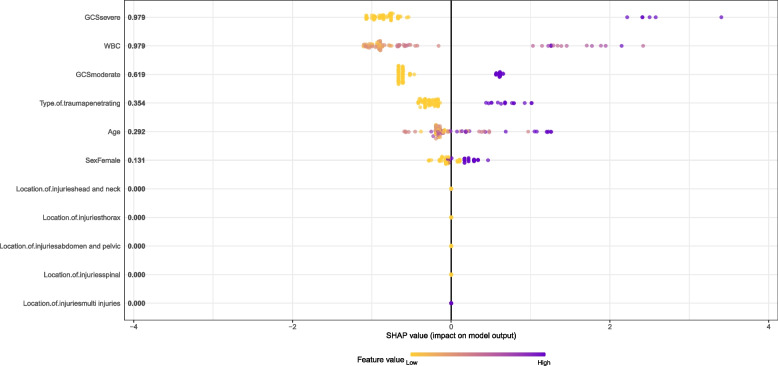
SHAP summary plot for input variables of the XGBoost model for predicting mortality traumatic injuries patients. WBC: white blood cells, GCS: Glasgow coma scale

To better understand the performance of the XGBoost model in predicting mortality and to identify the variables that influenced the prediction model, the SHAP summary plot was shown in Fig. 3. This plot indicates the ranking of variables' importance and the mean SHAP value. Positive SHAP values show that the model predicts patients with traumatic injuries who die, while negative SHAP values show patients with traumatic injuries who survive. SHAP values farther away from zero indicate a more impact for a specific variable.

Figure 3 demonstrates that the most important variables that have a significant impact on the prediction of the XGBoost model are GCS, WBC, type of trauma, age, and gender. In addition, it can be seen in Fig. 3 that the patients who died according to the prediction of the model had high values in all the important variables.

According to Figs. 2 and 3, the important variables detected in predicting trauma injury mortality with RF and XGBoost models were nearly identical.

## Discussion

In the current study, several machine learning methods were applied to predict traumatic injury outcomes in trauma patients referred to the Besat hospital of Hamadan province. Data in this study were highly imbalanced: approximately 7% of the people were classified as dead patients. The imbalance ratio was 13, which indicates that for each sample of the minority class (dead), there were 13 samples of the majority class (alive). Hence, we first used SMOTE balancing techniques for building balanced classes in the original dataset. These techniques are data oversampling approaches that are generally used more frequently than other approaches in studies and cause the improved performance of classifiers [29, 43, 47–51]. Then, machine learning methods were applied to predict the in-hospital mortality of patients with traumatic injuries.

In this regard, the six algorithms of machine learning, DT, RF, NB, ANN, SVM, and XGBoost, were constructed and evaluated to predict traumatic injury outcomes on balanced and imbalanced datasets. This study tried to show the undesirable impact of imbalanced data problems on the performance of the machine learning models and apply SMOTE balancing methods to solve them.

In general, the performance of machine learning methods based on the balanced datasets was remarkably better than that of models based on the original imbalanced dataset, as expected. This indicates to perform prediction using the SMOTE strategies on imbalanced data is rational.

The findings show a considerable difference between specificity and sensitivity in all of the used ML methods before applying to SMOTE methods, which indicates classifiers are biased toward the majority class. At the same time, there is little difference between the sensitivity and specificity of SMOTE-based machine learning algorithms. The slight difference between these two criteria was seen in other studies, too [48, 49, 52, 53].

Also, the evaluation results showed high accuracy for all ML methods except XGBoost before using SMOTE-balancing methods.

The main reason for achieving high accuracy in such a situation is that the classification algorithms are biased toward the majority class. Some studies have shown that when classes are imbalanced, the accuracy of classifiers is slightly higher than that of classifiers in balanced data [48–50]. However, some studies demonstrated a slight increase in the accuracy of classifiers with balanced data compared to imbalanced data [29, 51]. In the current study, a slight increase in the accuracy of classifiers with balanced data existed as compared to imbalanced data. Therefore, the accuracy criterion is not a sufficiently robust measure when facing imbalanced datasets classification problems. Hence, to evaluate ML algorithms' performance, the AUC criterion is widely used for evaluating classifiers in the imbalanced dataset [26].

The findings showed that the mean area under the ROC curve for all ML models in SMOTE-balanced datasets improved significantly compared with that in the imbalanced dataset. This accents the importance of using SMOTE balancing techniques.

Although the general performance of SMOTE-based machine learning algorithms is excellent, finding the appropriate SMOTE-balancing technique to get the best results from ML algorithms is tricky. There is no single SMOTE-balancing technique can achieve the best results for all ML algorithms.

The current study shows that ML algorithms work better on the data balanced by SMOTE-NC and SMOTE. Also, among all ML classifiers, ANN and RF models in SMOTE and the XGBoost model in SMOTE- NC outperformed other ML models.

It should be pointed out that was not possible to perform a comprehensive comparison in the present study for several reasons. First, there was no prior study conducted on the use of SMOTE-based ML algorithms in the trauma field that have focused on general trauma. However, these algorithms were employed in some fields. For example, Karajizadeh et al. had compared balancing approaches of under-sampling, oversampling, SMOTE, and ADASYN with SVM, ANN, C5.0 tree, and CHAID tree to predict in-hospital mortality from hospital-acquired infections in trauma patients. They reported that among these ML algorithms, the SVM algorithm by SMOTE balancing approach in terms of accuracy outperformed other ML algorithms by balancing approaches. The prediction accuracy by SVM with SMOTE was 100% [54]. Kumar et al. had also evaluated the performance of six ML algorithms: DT, k-Nearest Neighbor, Logistic regression, ANN, SVM, and NB over five imbalanced clinical datasets. They used seven balancing techniques for generating balanced data, namely under-sampling, random oversampling, SMOTE, ADASYN, SVM-SMOTE, SMOTEEN, and SMOTETOMEK. Then applied, ML algorithms were for the classification of balanced data. They reported that among seven balancing techniques, SMOTEEN had the best performance [29]. Second, there are many oversampling techniques in the field of imbalanced learning. So far, 85 oversampling techniques have been developed to solve the imbalanced data problem [26]. As a result, available studies used different SMOTE techniques that make comparison difficult and impossible. Third, the performance of both oversampling techniques and ML Models is generally data-dependent, one cannot detect an oversampling technique and ML classifier that always is the best for the classification of different datasets. Fourth, although various studies have investigated predicting trauma patient mortality using different ML methods. Nevertheless, most of these studies have concentrated on a specific type of trauma, such as burns, brain injuries, head injuries, and tooth injuries, and used the NN method [15–17, 55]. Hence, only a few studies were conducted in the trauma field focused on general trauma.

In this research, the RF model with SMOTE based on the evaluation criteria outperformed more ML methods. Consequently, the RF model has been used to identify the importance of variables in predicting traumatic injuries. The result of the variable importance based on the random forest model demonstrates that white blood cells and Glasgow coma scale and age, in terms of mean decrease accuracy and mean decrease Gini, have higher relative importance than other variables. Of these variables, WBC was identified as an important risk factor related to trauma mortality. This result is consistent with the findings of Almaghrabi et al. [47]. They compared the performance of DT, RF, ANN, SVM and Logistic regression to predict traumatic injury mortality and found all applied ML algorithms have similar prediction accuracy of 94%. However, based on AUC, logistic regression and RF have the highest value, and SVM has the lowest value. Also, the results of their study showed that the location of treatment and age are other important factors too.

External validation is critical for establishing ML algorithms' validity and reliability [56]. Therefore, there needs to be external validation attempts of SMOTE-based ML algorithms using an alternative external dataset. Therefore, the lack of external validation in our current study is one of the limitations.

Another limitation of the present study is that the data employed here were obtained from a registry-based retrospective study which causes the analysis to be prone to potential biases for the estimations for measures such as sensitivity. In addition, our study had a small sample size. Therefore, studies with large sample sizes are needed to investigate the performance and reliability of these methods. Also, factors such as injury severity scale (ISS), vital signs, and infection need to be considered in future predictive models in these patients.

Recently, to overcome the limitations of SMOTE, new versions of SMOTE have been introduced. Therefore, the authors propose to use the new versions of SMOTE, e.g., A-SMOTE, RN-SMOTE, SMOTE-LOF, to deal with imbalances and compare them with the prior versions of SMOTE for further analysis [28, 57, 58].

In this study, we used SMOTE and modifications of SMOTE to account for borderline samples in the classification of imbalanced datasets. In future work, we will use variants of SMOTE to detect noise samples. We will also employ deep learning methods to detect noise and borderline samples and to resample data.

## Conclusion

Prediction models are broadly used in healthcare management, medical sciences, and clinical decision support. These methods help identify the rate of patient injuries, prioritize immediate threats, and decision-making in trauma. Hence causes improved medical care and the development of trauma services. Prediction models can help ICU physicians determine which patients are at high risk of mortality and who should be prioritized for treatment, enabling them to optimize clinical interventions and improve patients' prognoses. According to the excellent performance of machine learning models based on the SOMTE technique in predicting mortality in this study, the design of accurate decision support systems using these models facilitates and accelerates healthcare management processes.

Our finding demonstrated that RF and ANN models with SOMTE and XGBoost model with SMOTE-NC may be better than other ML models in predicting traumatic injury outcomes in trauma patients in terms of all criteria. Also, the most important variable affecting the predicting mortality in trauma patients based on SHAP value and RF were the white blood cells, the Glasgow coma scale, and age. However, these results are based on the finding of our study and do not have a generalization ability. Consequently, simulation studies are suggested for more investigation. Simulation studies are needed to investigate overall results and recommend a valuable tool for hospital mortality prediction in patients with traumatic injuries.

## Supplementary Information

**Additional file 1: Table A1.** Comparison of the predictive performance of SMOTE-based machine learning methods in terms of 95% confidence intervals of the evaluation criteria on a test data set.

**Additional file 2: Figure A1.** The performance comparison of classifiers with SMOTE techniques and without SMOTE in terms of sensitivity, Specificity. **Figure A2.** The performance comparison of classifiers with SMOTE techniques and without SMOTE in terms of Positive Predictive Value and Negative Predictive Value.

## Data Availability

The dataset used for analysis during the current study is not publicly available due to restrictions related to our internal review board policy. However, the dataset is available from the corresponding author upon reasonable request.

## Abbreviations

DT: Decision Tree
RF: Random Forest
NB: Naïve Bayes
ANN: Artificial Neural Network
SVM: Support Vector Machine
XGBoost: Extreme Gradient Boosting
SHAP: SHapley Additive exPlanations
PPV: Positive Predictive Value
NPV: Negative Predictive Value
AUC: Area Under the Curve
SMOTE: Synthetic Minority Over-Sampling Technique
SVM-SMOTE: Support Vector Machine Synthetic Minority Over-Sampling Technique
SMOTE-NC: Synthetic Minority Over-Sampling Technique- Nominal Continuous
LOS: Length of Stay in ICU
WBC: White Blood Cells
GCS: Glasgow Coma Scale
