# Supplementary material for: Hospital mortality prediction in traumatic injuries patients: comparing different SMOTE-based machine learning algorithms
Source: BMC Med Res Methodol. 2023 Apr 22;23:101. doi: 10.1186/s12874-023-01920-w (PMC10122327; doi:10.1186/s12874-023-01920-w)
Supplement: Supplementary file 1 — Additional file 1: Table A1. Comparison of the predictive performance of SMOTE-based machine learning methods in terms of 95% confidence intervals of the evaluation criteria on a test data set. [file 12874_2023_1920_MOESM1_ESM.docx]

Table A1. Comparison of the predictive performance of SMOTE-based machine learning methods in terms of 95% confidence intervals of the evaluation criteria on a test data set.

| **Methods** | **Dataset** | **Sensitivity** |  | **Specificity** |  | **PPV** |  | **NPV** |  | **Accuracy** |  | **AUC** |  | **G-means** |  | **F1-Score** |  | **McNemar** |
| --- | --- | --- | --- | --- | --- | --- | --- | --- | --- | --- | --- | --- | --- | --- | --- | --- | --- | --- |
|  |  | **(95% CI)** |  | **(95% CI)** |  | **(95% CI)** |  | **(95% CI)** |  | **(95% CI)** |  | **(95% CI)** |  | **(95% CI)** |  | **(95% CI)** |  | **(95% CI)** |
| **SVM** | Original | (0.458, 0.642) |  | **-** |  | **-** |  | (0.953, 0.967) |  | (0.963, 0.977) |  | (0.701, 0.739) |  | (0.631, 0.689) |  | (0.562, 0.638) |  | (0.718, 0.782) |
|  | SMOTE | (0.978, 0.981) |  | (0.966, 0.974) |  | (0.966, 0.974) |  | **(0.989, 0.990)** |  | (0.988, 0.992) |  | (0.978, 0.981) |  | (0.978, 0.981) |  | (0.978, 0.981) |  | (0.211, 0.309) |
|  | B-SMOTE1 | **(0.988, 0.992)** |  | (0.956, 0.964) |  | (0.956, 0.964) |  | (0.987, 0.993) |  | (0.978, 0.982) |  | (0.978, 0.982) |  | (0.978, 0.982) |  | (0.978, 0.982) |  | (0.721, 0.759) |
|  | B-SMOTE2 | (0.943, 0.957) |  | (0.954, 0.966) |  | (0.954, 0.966) |  | (0.943, 0.957) |  | (0.946, 0.954) |  | (0.946, 0.954) |  | (0.946, 0.954) |  | (0.946, 0.954) |  | (0.467, 0.593) |
|  | SMOTE-NC | (0.979, 0.981) |  | (0.989, 0.990) |  | (0.989, 0.990) |  | (0.979, 0.981) |  | **(0.989, 0.991)** |  | **(0.989, 0.990)** |  | **(0.989, 0.990)** |  | **(0.989, 0.990)** |  | (0.311, 0.349) |
|  | SVM-SMOTE | (0.988, 0.992) |  | (0.955, 0.965) |  | (0.932, 0.948) |  | (0.988, 0.992) |  | (0.977, 0.983) |  | (0.978, 0.982) |  | (0.978, 0.982) |  | (0.966, 0.974) |  | (0.706, 0.754) |
|  |  |  |  |  |  |  |  |  |  |  |  |  |  |  |  |  |  |  |
| **ANN** | Original | (-0.685, 2.245) |  | **-** |  | (0.720, 0.800) |  | (0.750, 0.770) |  | (0.937, 1.023) |  | (0.748, 1.212) |  | (0.766, 0.854) |  | (0.642, 0.738) |  | (0.100, 0.420) |
|  | SMOTE | **-** |  | **-** |  | **-** |  | **-** |  | **-** |  | **-** |  | **-** |  | **-** |  | - |
|  | B-SMOTE1 | **-** |  | **-** |  | **-** |  | **-** |  | **-** |  | **-** |  | **-** |  | **-** |  | - |
|  | B-SMOTE2 | (0.977, 0.983) |  | (0.976, 0.984) |  | (0.976, 0.984) |  | (0.977, 0.983) |  | (0.977, 0.983) |  | (0.978, 0.982) |  | (0.978, 0.982) |  | (0.977, 0.983) |  | (0.161, 0.259) |
|  | SMOTE-NC | (0.967, 0.973) |  | (0.978, 0.982) |  | (0.977, 0.983) |  | (0.977, 0.983) |  | (0.987, 0.993) |  | (0.978, 0.982) |  | (0.978, 0.982) |  | (0.977, 0.983) |  | (0.155, 0.245) |
|  | SVM-SMOTE | (0.979, 0.981) |  | (0.989, 0.990) |  | (0.979, 0.981) |  | (0.989, 0.990) |  | (0.989, 0.990) |  | (0.979, 0.980) |  | (0.979, 0.980) |  | (0.979, 0.981) |  | (0.324, 0.336) |
|  |  |  |  |  |  |  |  |  |  |  |  |  |  |  |  |  |  |  |
| **NB** | Original | (0.326, 0.694) |  | (0.941, 0.959) |  | (0.356, 0.484) |  | (0.953, 0.967) |  | (0.897, 0.923) |  | (0.653, 0.747) |  | (0.627, 0.693) |  | (0.403, 0.477) |  | (0.464, 0.596) |
|  | SMOTE | **(0.989, 0.991)** |  | (0.900, 0.920) |  | (0.911, 0.929) |  | **(0.989, 0.991)** |  | (0.945, 0.955) |  | (0.945, 0.955) |  | (0.945, 0.955) |  | (0.955, 0.965) |  | (0.841, 0.899) |
|  | B-SMOTE1 | (0.988, 0.992) |  | (0.911, 0.929) |  | (0.922, 0.938) |  | (0.987, 0.993) |  | (0.955, 0.965) |  | (0.955, 0.965) |  | (0.955, 0.965) |  | (0.955, 0.965) |  | (0.828, 0.872) |
|  | B-SMOTE2 | (0.953, 0.967) |  | (0.848, 0.872) |  | (0.859, 0.881) |  | (0.952, 0.968) |  | (0.903, 0.917) |  | (0.903, 0.917) |  | (0.903, 0.917) |  | (0.903, 0.917) |  | (0.693, 0.807) |
|  | SMOTE-NC | (0.976, 0.984) |  | **(0.944, 0.956)** |  | **(0.944, 0.956)** |  | (0.976, 0.984) |  | **(0.956, 0.964)** |  | **(0.956, 0.964)** |  | **(0.956, 0.964)** |  | **(0.956, 0.964)** |  | (0.555, 0.665) |
|  | SVM-SMOTE | (0.988, 0.992) |  | (0.900, 0.920) |  | (0.868, 0.892) |  | (0.975, 1.005) |  | (0.934, 0.946) |  | (0.945, 0.955) |  | (0.945, 0.955) |  | (0.923, 0.937) |  | (0.835, 0.885) |
|  |  |  |  |  |  |  |  |  |  |  |  |  |  |  |  |  |  |  |
| **RF** | Original | (0.462, 0.598) |  | (0.989, 0.991) |  | (0.936, 1.024) |  | (0.942, 0.958) |  | (0.952, 0.968) |  | (0.686, 0.734) |  | (0.616, 0.684) |  | (0.556, 0.644) |  | (0.715, 0.805) |
|  | SMOTE | **(0.989, 0.991)** |  | **(0.989, 0.990)** |  | **(0.989, 0.990)** |  | **(0.989, 0.991)** |  | **(0.989, 0.991)** |  | **(0.989, 0.990)** |  | **(0.989, 0.990)** |  | **(0.989, 0.990)** |  | (0.058, 0.082) |
|  | B-SMOTE1 | (0.979, 0.981) |  | (0.989, 0.990) |  | (0.989, 0.990) |  | (0.978, 0.982) |  | (0.989, 0.991) |  | (0.979, 0.981) |  | (0.989, 0.991) |  | (0.989, 0.990) |  | (0.059, 0.101) |
|  | B-SMOTE2 | (0.965, 0.975) |  | (0.976, 0.984) |  | (0.965, 0.975) |  | (0.965, 0.975) |  | (0.976, 0.983) |  | (0.967, 0.973) |  | (0.965, 0.973) |  | (0.967, 0.973) |  | (0.323, 0.437) |
|  | SMOTE-NC | (0.967, 0.973) |  | (0.977, 0.983) |  | (0.977, 0.983) |  | (0.967, 0.973) |  | (0.987, 0.993) |  | (0.978, 0.982) |  | (0.978, 0.982) |  | (0.977, 0.983) |  | (0.200, 0.300) |
|  | SVM-SMOTE | (0.966, 0.974) |  | (0.989, 0.990) |  | (0.979, 0.980) |  | (0.978, 0.982) |  | (0.988, 0.992) |  | (0.978, 0.982) |  | (0.978, 0.982) |  | (0.968, 0.972) |  | (0.217, 0.283) |
|  |  |  |  |  |  |  |  |  |  |  |  |  |  |  |  |  |  |  |
| **DT** | Original | (-0.877, 1.397) |  | (0.931, 0.949) |  | (0.082, 0.198) |  | (0.951, 0.969) |  | (0.888, 0.912) |  | (0.545, 0.655) |  | (0.424, 0.476) |  | (0.233, 0.267) |  | (0.236, 0.464) |
|  | SMOTE | (0.928, 0.952) |  | (0.909, 0.931) |  | (0.909, 0.931) |  | (0.930, 0.950) |  | (0.923, 0.937) |  | (0.923, 0.937) |  | (0.923, 0.937) |  | (0.923, 0.937) |  | (0.587, 0.713) |
|  | B-SMOTE1 | (0.921, 0.939) |  | (0.919, 0.941) |  | (0.919, 0.941) |  | (0.921, 0.939) |  | (0.924, 0.936) |  | (0.924, 0.936) |  | (0.924, 0.936) |  | (0.924, 0.936) |  | (0.581, 0.699) |
|  | B-SMOTE2 | (0.907, 0.933) |  | (0.895, 0.925) |  | (0.896, 0.924) |  | (0.918, 0.942) |  | (0.904, 0.916) |  | (0.915, 0.925) |  | (0.904, 0.916) |  | (0.904, 0.916) |  | (0.772, 0.868) |
|  | SMOTE-NC | (0.932, 0.948) |  | **(0.961, 0.979)** |  | **(0.961, 0.979)** |  | (0.943, 0.957) |  | **(0.955, 0.965)** |  | **(0.955, 0.965)** |  | **(0.955, 0.965)** |  | **(0.955, 0.965)** |  | (0.791, 0.849) |
|  | SVM-SMOTE | **(0.942, 0.958)** |  | (0.928, 0.952) |  | (0.894, 0.926) |  | **(0.964, 0.976)** |  | (0.932, 0.948) |  | (0.933, 0.947) |  | (0.933, 0.947) |  | (0.921, 0.939) |  | (0.519, 0.641) |
|  |  |  |  |  |  |  |  |  |  |  |  |  |  |  |  |  |  |  |
| **XGBoost** | Original | (0.985, 0.995) |  | (0.426, 0.474) |  | (0.116, 0.144) |  | (0.989, 0.991) |  | (0.467, 0.513) |  | (0.600, 0.840) |  | (0.639, 0.681) |  | (0.199, 0.241) |  | (0.989, 0.991) |
|  | SMOTE | (0.986, 0.994) |  | (0.952, 0.968) |  | (0.952, 0.968) |  | (0.976, 0.984) |  | (0.966, 0.974) |  | (0.966, 0.974) |  | (0.966, 0.974) |  | (0.966, 0.974) |  | (0.571, 0.709) |
|  | B-SMOTE1 | (0.975, 0.985) |  | (0.952, 0.968) |  | (0.952, 0.968) |  | (0.974, 0.986) |  | (0.966, 0.974) |  | (0.966, 0.974) |  | (0.966, 0.974) |  | (0.966, 0.974) |  | (0.569, 0.711) |
|  | B-SMOTE2 | (0.954, 0.966) |  | (0.932, 0.948) |  | (0.932, 0.948) |  | (0.954, 0.966) |  | (0.946, 0.954) |  | (0.946, 0.954) |  | (0.946, 0.954) |  | (0.945, 0.955) |  | (0.538, 0.662) |
|  | SMOTE-NC | **-** |  | **-** |  | **-** |  | **-** |  | **-** |  | **-** |  | **-** |  | **-** |  | - |
|  | SVM-SMOTE | (0.959, 0.981) |  | (0.951, 0.969) |  | (0.928, 0.952) |  | (0.973, 0.987) |  | (0.954, 0.966) |  | (0.954, 0.966) |  | (0.954, 0.966) |  | (0.943, 0.957) |  | (0.607, 0.733) |

SVM: support vector machine, ANN: artificial neural network, NB: naïve Bayes, RF: random forest, DT: Decision Tree, XGBoost: Extreme Gradient Boosting, SMOTE: synthetic minority over-sampling technique, B-SMOTE1: Borderline-SMOTE1, B-SMOTE-2: Borderline-SMOTE2, PPV: positive predictive value, NPV: negative predictive value, AUC: area under the curve, CI: confidence interval
