# Supplementary material for: Hospital mortality prediction in traumatic injuries patients: comparing different SMOTE-based machine learning algorithms
Source: BMC Med Res Methodol. 2023 Apr 22;23:101. doi: 10.1186/s12874-023-01920-w (PMC10122327; doi:10.1186/s12874-023-01920-w)
Supplement: Supplementary file 2 — Additional file 2: Figure A1. The performance comparison of classifiers with SMOTE techniques and without SMOTE in terms of sensitivity, Specificity. Figure A2. The performance comparison of classifiers with SMOTE techniques and without SMOTE in terms of Positive Predictive Value and Negative Predictive Value. [file 12874_2023_1920_MOESM2_ESM.docx]

| **Sensitivity**   |
| --- |
| **Specificity**    Figure A1. The performance comparison of classifiers with SMOTE techniques and without SMOTE in terms of sensitivity, Specificity. |

| **Positive Predictive Value**   |
| --- |
| **Negative Predictive Value**    Figure A2. The performance comparison of classifiers with SMOTE techniques and without SMOTE in terms of Positive Predictive Value and Negative Predictive Value. |
